# Visualizing stellate ganglion with US imaging for guided SGB treatment: A feasibility study with healthy adults

**DOI:** 10.3389/fnins.2022.998937

**Published:** 2022-09-09

**Authors:** Jia Li, Shaofeng Pu, Zihao Liu, Lixin Jiang, Yuanyi Zheng

**Affiliations:** ^1^Department of Ultrasound in Medicine, Sixth People's Hospital affiliated to Shanghai Jiao Tong University, Shanghai, China; ^2^Department of Pain Management, Sixth People's Hospital affiliated to Shanghai Jiao Tong University, Shanghai, China; ^3^Department of Ultrasound, Renji Hospital, School of Medicine, Shanghai Jiao Tong University, Shanghai, China

**Keywords:** stellate ganglion, ultrasound, cervical sympathetic ganglion, cervical sympathetic trunk, ganglion block

## Abstract

**Objective:**

As for ultrasound (US) guided stellate ganglion (SG) block, unsatisfactory curative outcomes and complications still remain. This problem could be greatly improved by identifying and monitoring SG. To the best of our knowledge, there are few reports to directly visualize SG in literature. This study explored the feasibility of detection of SG and summarized the findings of SG through US.

**Methods:**

Fifty healthy adults with 100 SGs were enrolled. The size, shape, echogenicity, margin, the inferior pole of SG, the relationship between the superior pole of SG and the transverse process, the relationship between the superior pole of SG and the inferior thyroid artery, and the relationships between SG and other surrounding tissues were evaluated by US.

**Results:**

The SG was identified in 79% of the participants. No significant differences were found between the right and left sides regarding thickness, cross-sectional area (CSA), and position (all *p* > 0.05); however, there was a significant difference in the width of the right and left sides (*p* < 0.05). Side was associated with SG visibility (*p* < 0.05), however, the gender was not (*p* > 0.05). A total of 42% of SGs were oval-shaped. All SGs were hyperechogenic and had an ill-defined margin. In fact, 63% of SGs were located in the C7 transverse process level, 77% of SGs were located under the inferior thyroid artery, and all of these SGs were located lateral to the thyroid and medial to the anterior scalene muscle and the vagus nerve.

**Conclusion:**

Our preliminary study demonstrates that US imaging provides the capability of detecting SG. This may be helpful in minimizing complications and improving the accuracy of US-guided SG block.

## Introduction

Stellate ganglion block (SGB) is frequently used for the treatment of many kinds of medical conditions by injecting a local anesthetic around stellate ganglion (SG) in clinical practice. It influences both the central and peripheral nervous systems. The former mainly influences the hypothalamus, whose function is to regulate the systemic autonomic nervous, immune, and endocrine systems and to maintain homeostasis and normal cardiovascular function (Yokoyama et al., 2000). The latter regulates vascular dilatation and constriction, muscular movement, bronchial smooth muscle relaxation and contraction, and pain conduction (Chen et al., 2016), of which sympathetically mediated pain affecting the head, neck, and upper extremities is a routine procedure (Piraccini et al., 2022). Currently, it is prospective to apply SGB to treat cardiac arrhythmia (Tian et al., 2019), post-traumatic stress disorder (Lipov et al., 2012; Mulvaney et al., 2014), menopause, and hot flashes related to cancer therapy (Othman and Zaky, 2014; Rahimzadeh et al., 2018). Also, SGB under US guidance is beneficial for the recovery of gastrointestinal functions and the reduction of stress responses in patients with colorectal cancer on whom laparoscopic colorectal surgery had been performed (Zhu et al., 2021). Although the effectiveness of SGB is well-established, complications may be caused during the procedure due to various vital structures around the SG. In addition, some patients fail to improve and resolve symptoms.

With efforts to increase the safety and efficacy concerning SGB, the methods have developed over time, from traditional blind technique to fluoroscopy-guided technique to computerized tomography (CT), magnetic resonance imaging (MRI), and recently ultrasound (US) guided-technique. Initially, a blind technique is performed according to the knowledge of anatomical landmarks. However, due to the uncertainty of the needle position and drug diffusion, this classical approach can result in various complications, including intravascular injection, recurrent laryngeal injury, formation of hematomas, esophageal injury, and even death (Elias, 2000; Narouze et al., 2007; Gofeld et al., 2009). The fluoroscopy guidance, which is performed by injecting a contrast agent, is safer and more effective than the blind technique (Elias, 2000; Abdi et al., 2004), because it provides excellent views of body surfaces, and this is helpful for recognizing the transverse processes. However, similar to the blind technique, fluoroscopy guidance also cannot visualize the soft tissues, and thus these structure injuries are possible. In most cases, CT and MRI guidance are impractical in clinical settings, because they are believed to be costly and time consuming. In addition, CT guidance has the disadvantage of radiation exposure. Currently, in comparison with blind technique and fluoroscopy guidance, US guidance has become the preferred approach in terms of SGB. As a reliable imaging approach, US guidance can greatly promote safety and efficacy by direct visualization of the soft tissues, bony surfaces, and dynamic observation of the needle path and the spread of drugs. SGB under US guidance is more accurate and greatly reduces complications than the blind technique (Ding et al., 2017; Elmofty and Eckmann, 2019). However, US guidance still has unresolved issues regarding safety and efficacy. Some studies reported that complications, such as hoarseness, arrhythmia, dysphagia, hematoma, swallowing difficulty, foreign body sensation, and upper extremity weakness, still existed in a small group of patients with US guidance (Jung et al., 2011; Aleanakian et al., 2020; Shan et al., 2022). With respect to efficacy, for example, Aleanakian et al. (2020) reported that 27% of patients had no reduction of spontaneous pain by SGB under US guidance. Jung et al. (2011) demonstrated that nine patients undergoing US-guided SGB were not successfully blocked. The problem may be attributed, in part, to SG variations, large volumes of local anesthetics, and inadequate spread of drugs to SG.

If the SG itself can be directly visualized and monitored during the procedure, the above problems could be greatly improved, thus increasing safety and efficacy. Nevertheless, so far, US-guided SGB has been performed by identifying the surrounding tissues of the SG, including the prevertebral fascia and the longus colli muscle (LCM) (McDonnell et al., 2011; Bhatia et al., 2012; Soneji and Peng, 2013). There are few reports to directly visualize SG itself before US-guided SGB in the clinic. Therefore, the purpose of this study was (1) to explore the feasibility of US detection of SG, and (2) to summarize the US findings on SG.

## Materials and methods

### Patient

**Figure 1:**
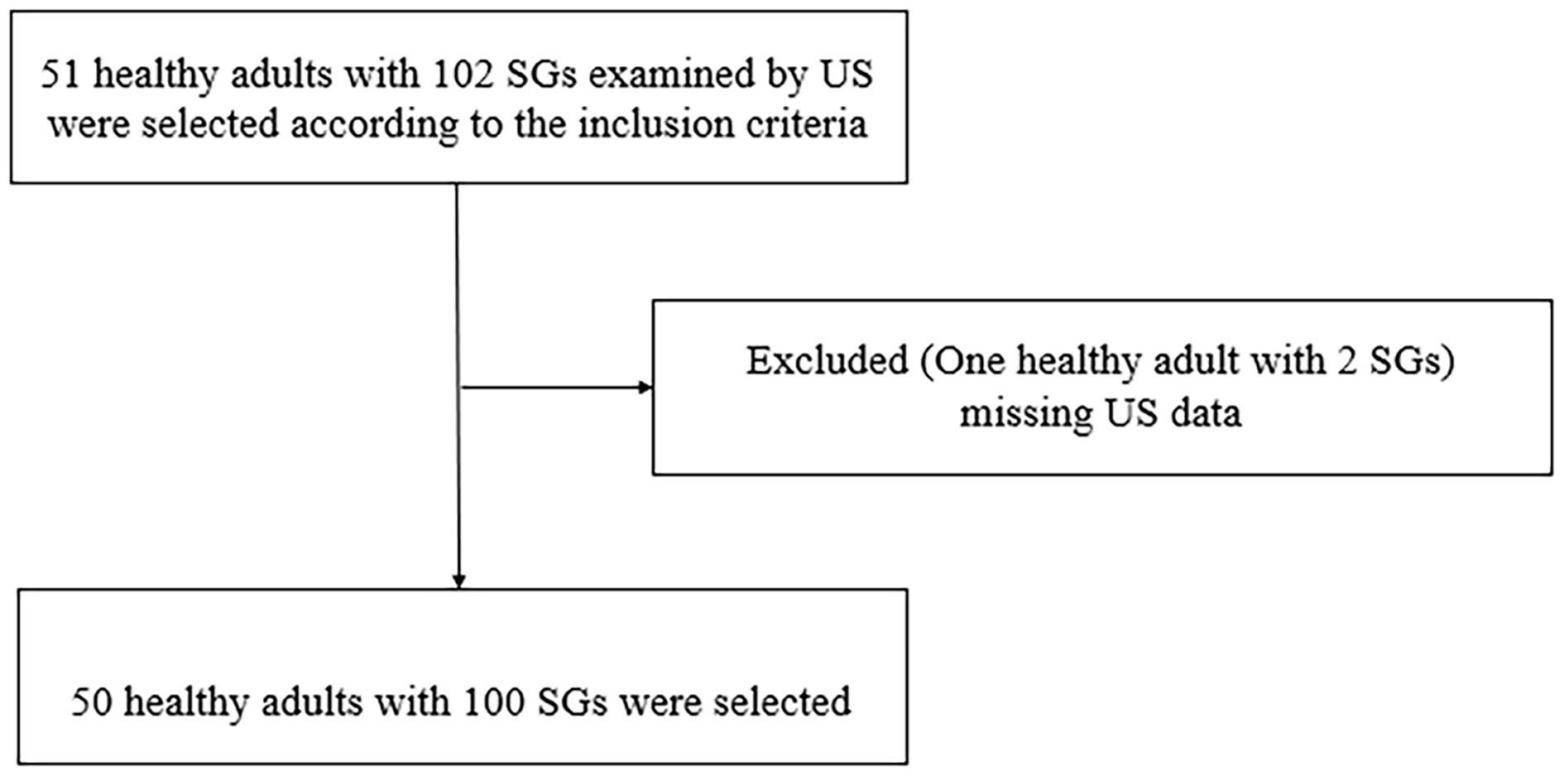
The flow diagram of the study population.

This retrospective study was approved by the Institutional Ethics Committee. Informed written consent was obtained from each participant. Fifty-one healthy adults with 102 SGs were selected in the Shanghai Jiao Tong University Affiliated Sixth People's Hospital, between June and October 2021. One healthy adult with two SGs was excluded because of missing US data, and 100 SGs were selected. There were 10 men and 40 women, with an average age of 27.22 ± 5.46 years (range 23–56 years). The inclusion criteria were age ≥18 years, with no history of neck irradiation and neck surgery or trauma. Figure 1 presents the flow diagram of the study population.

### SG identification

The cervical sympathetic chain, including SG, is usually located posteromedial to the carotid sheath and anterior to the LCM, between the C7 transverse process and the first rib (Civelek et al., 2008; Won et al., 2020). Depending on the figure of cadaveric studies, SG appears as a local nodule in the cervical sympathetic nerve in comparison with the cervical sympathetic trunk (Kiray et al., 2005; Won et al., 2020). Ultrasound has some limitations in identifying the first rib from the second rib. While the first rib is adjacent to the supraclavicular fossa, it could be identified easily. Therefore, we chose the supraclavicular fossa as the landmark on US images. The visibility of SG on the US needs to meet the following conditions: the nodular structure; between the carotid sheath and the LCM; and between the C7 transverse process and the supraclavicular fossa. At the same time, to increase the accuracy of detection, SG should be visible on both the transverse section and longitudinal section.

### US examination

All US examinations were performed by a sonographer using an 18 MHz linear-array transducer (Toshiba Aplio 500, Japan). The participants were asked to lie in the supine position and to slightly turn their necks to the opposite side, while relaxing their necks. At first, the ultrasound transducer was placed on the transverse section and scanned from up to down, up to the C7 transverse process, and down to the supraclavicular fossa. Looking for the SG between the carotid sheath and the LCM. Afterward, the transducer was rotated 90° to acquire the longitudinal section of the SG. If SG was found on both transverse and longitudinal sections, then the width, thickness, cross-sectional area (CSA), length, shape, echogenicity, margin, the inferior pole of SG, the relationship between the superior pole of SG and the transverse process, and the relationship between the superior pole of SG and the inferior thyroid artery were evaluated using US. In addition, the relationships between SG and other surrounding tissues, such as thyroid, anterior scalene muscle, and vagus nerve, were also evaluated. The width, thickness, and CSA were measured on the transverse section. The CSA was measured by tracing a continuous line. The length was measured in the longitudinal section. The shape was also evaluated in the longitudinal section. It comprises star, spindle, oval, triangular, and dumbbell forms. The inferior pole of SG was visible or invisible. Compared to LCM, echogenicity includes hyperechoic, isoechoic and hypoechoic features. The margin showed well-defined or ill-defined. The relationships of the superior pole of SG to the inferior thyroid artery were divided into three categories, that is, under, at the same level, and not assessed. Regarding the first type, the distance of the superior pole of SG to the inferior thyroid artery was measured.

### Statistical analysis

Statistical analysis was conducted by SPSS 19 statistical software. Continuous variables were calculated as mean ± standard deviation (SD). Counting data were expressed as the number (%). The independent *t*-test was applied to compare differences in width, thickness, CSA, and length between the right and left sides (null hypothesis: no width, thickness, CSA, and length difference). The χ^2^-test was applied to evaluate differences in the position between the right and left sides. The χ^2^-test was also used for assessing the relationship between sex, side (right and left), and visualization of SG. All *p*-values were two-sided. A value of *p* < 0.05 was defined as statistically significant.

## Results

In fifty participants with 100 SGs, the SG was identified in 79% (79/100) of the participants. The right SG was identified in 66% (33/50), and the left SG was identified in 92% (46/50). One participant was not identified on the right or left side. Of the remaining 49 participants, 30 participants (61%) were identified bilaterally and 19 participants (39%) were identified unilaterally (right, 16; left, 3).

The mean width, thickness, CSA, and length measurements of SG were 5.42±0.95 mm (range, 3.6–7.7 mm), 3.32 ± 0.77 mm (range, 1.8–5.3 mm), 14.08 ± 4.42 mm^2^ (range, 5–27 mm^2^), and 18.10 ± 3.90 mm (9.8–25.2 mm), respectively. Among the 79 SGs, the lengths of 67 SGs were calculated because the inferior poles of the remaining SGs were not visible. The mean width, thickness, CSA, and length measurements for the right and left sides are summarized in Table 1. No significant differences were found between the right and left regarding thickness, CSA, and position (all *p* > 0.05). Nevertheless, there was a significant difference in the width of the right and left sides (*p* < 0.05). The right width was significantly higher than the left. In addition, the side was associated with SG visibility (*p* < 0.05); however, the gender was not (*p* > 0.05; Table 2).

**Table 1:** Comparison of US findings between the right and left sides.

**US findings**	**Right (*n* = 33)**	**Left (*n* = 46)**	***P-*value**
Width (mm)	5.83 ± 1.08	5.13 ± 0.74	0.020^*^
Thickness (mm)	3.04 ± 0.88	3.52 ± 0.61	0.080
CSA (mm^2^)	14.18 ± 5.15	14 ± 3.88	0.865
Length (mm)	17.54 ± 3.83^†^	18.53 ± 3.95^‡^	0.308
**Position**			0.598
C7 transverse process level	22 (67%)	28 (61%)	
behind C7 transverse process level	11 (33%)	18 (39%)	

CSA, cross-sectional area.

Data are presented as mean ± standard deviation or number (present).

† 29 SGs.

‡ 38 SGs.

* Statistically significant at a p-value < 0.05.

**Table 2:** Factors related to SG visibility.

	**Visibility**		
	**Detected**	**Not detected**	***P-*value**
	**(*n* = 79)**	**(*n* = 21)**	
**Patient sex**			1.000
Men	16	4	
Women	63	17	
**Side**			0.001^*^
Right	33	17	
Left	46	4	

* Statistically significant at a p-value < 0.05.

**Figure 2:**
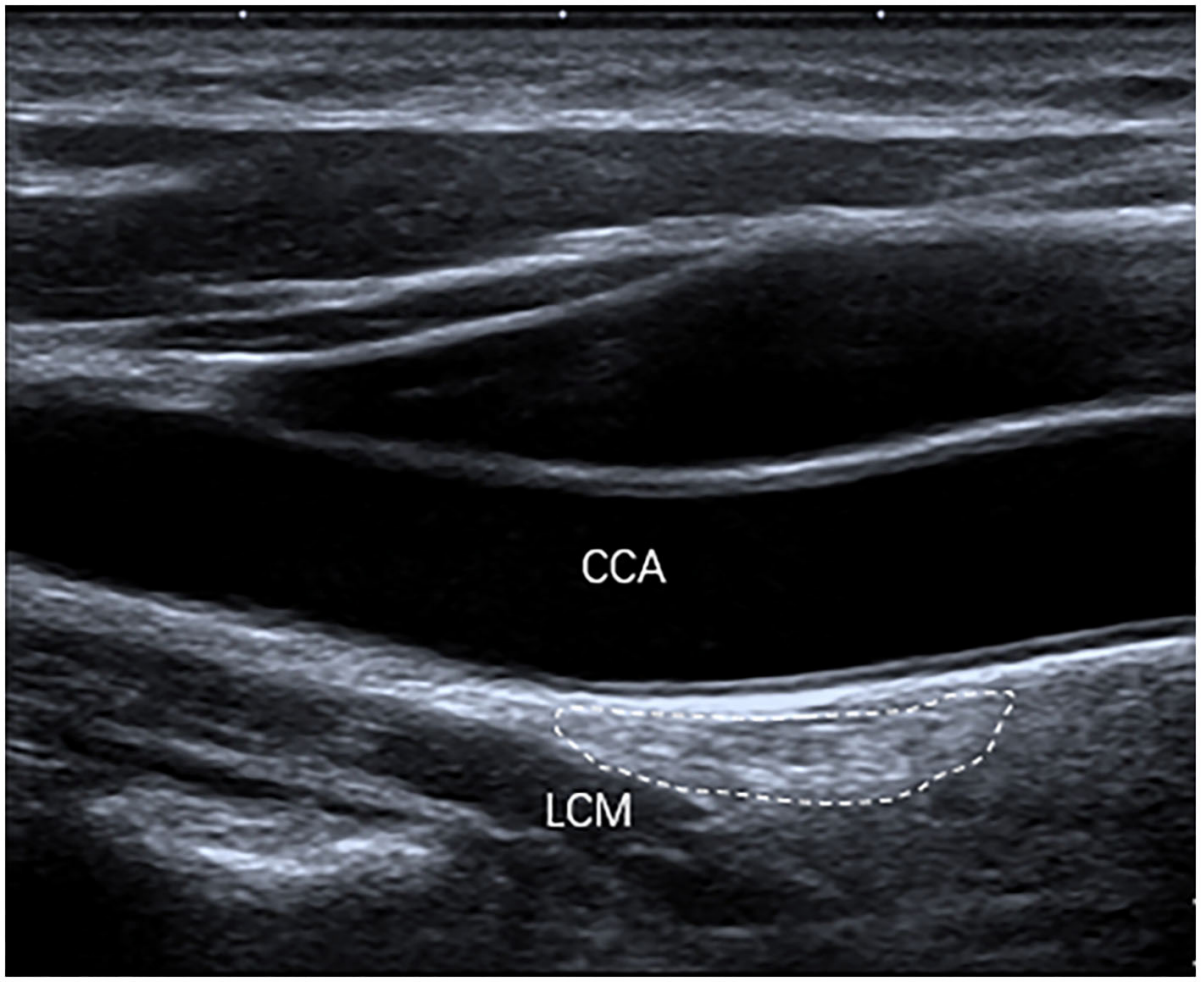
The SG, with an oval shape (outlined) and hyperechogenicity, was located between the common carotid artery (CCA) and the longus colli muscle (LCM) on the longitudinal ultrasound image.

**Figure 3:**
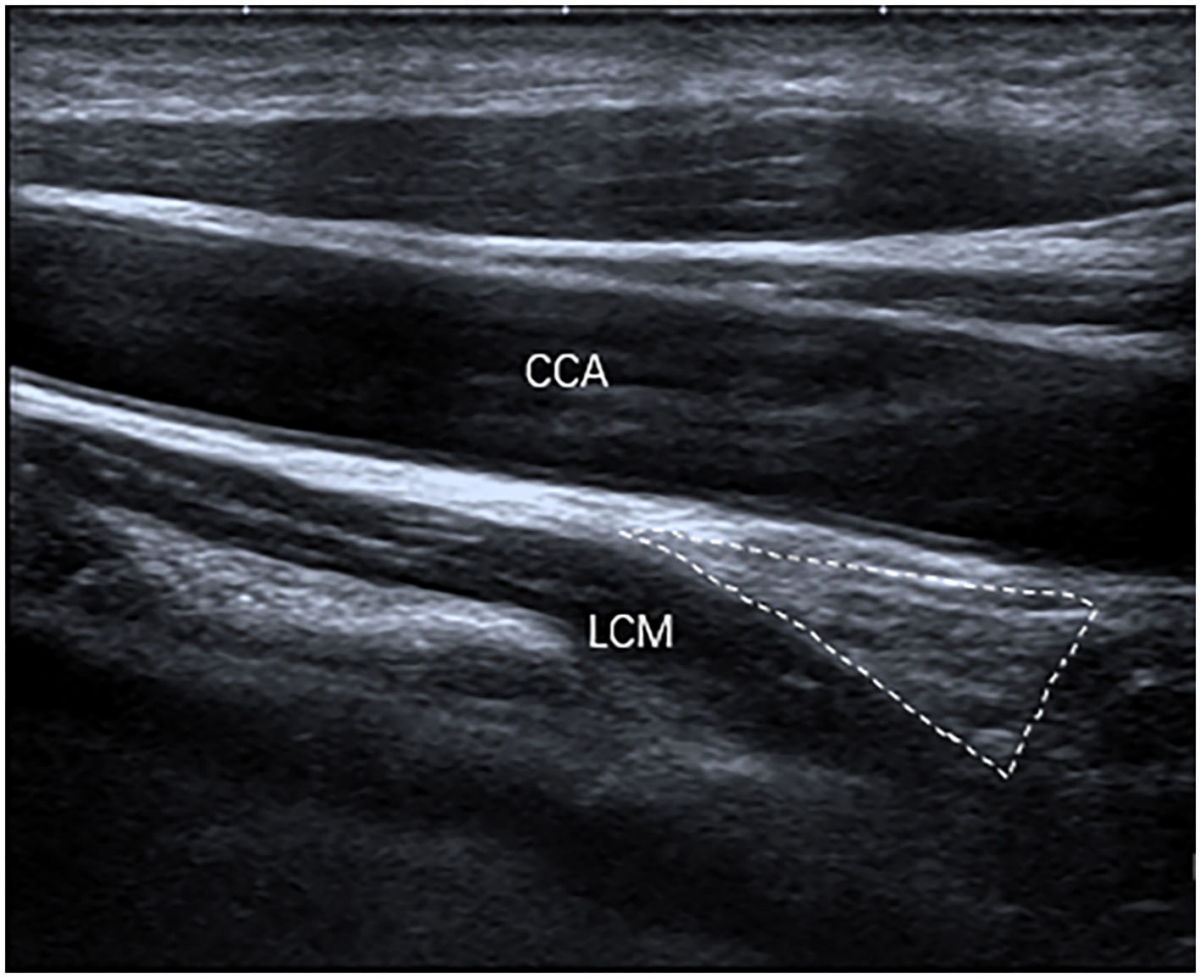
The SG, with a triangular shape (outlined) and hyperechogenicity, was located between the common carotid artery (CCA) and the longus colli muscle (LCM) on the longitudinal ultrasound image.

**Figure 4:**
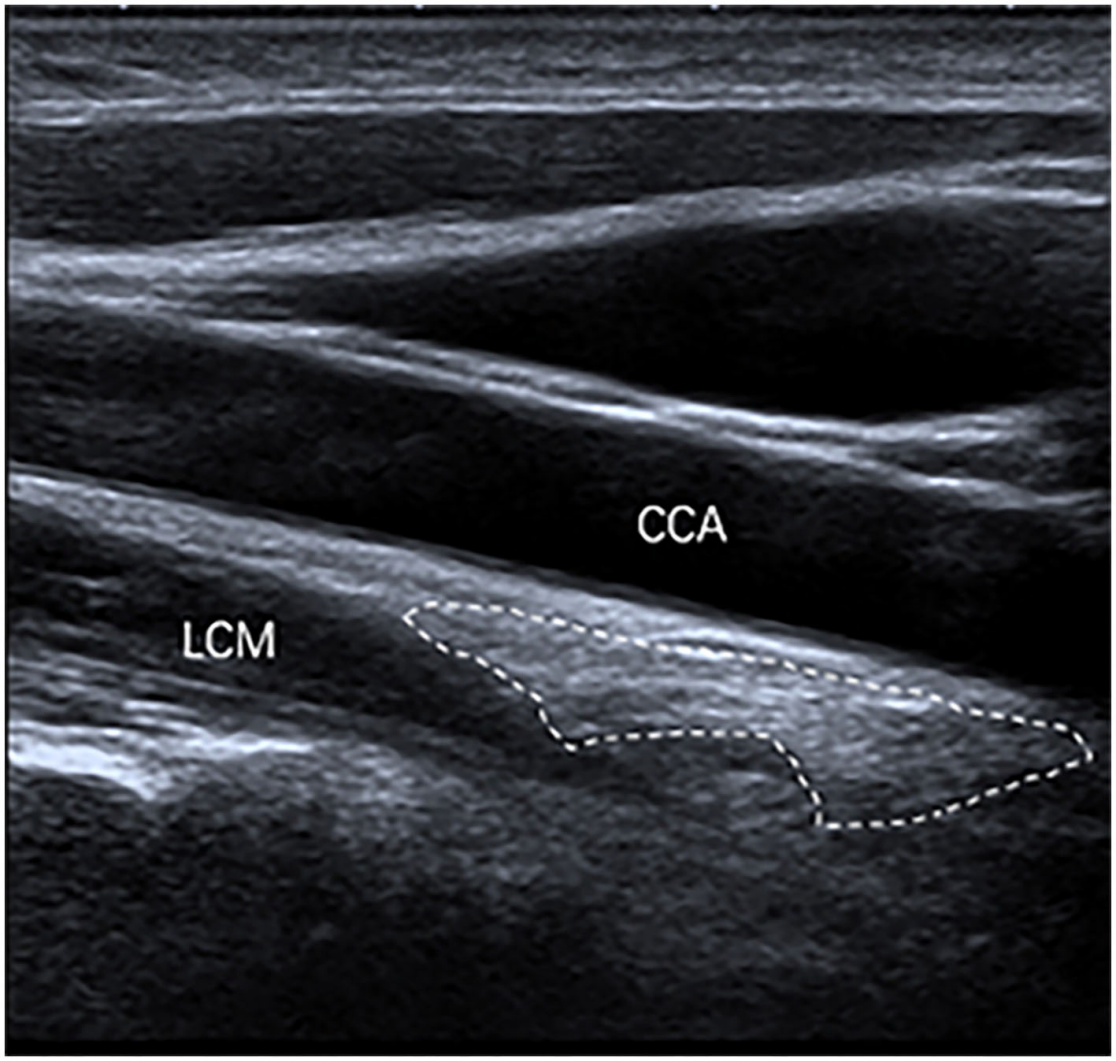
The SG, with a dumbbell shape (outlined) and hyperechogenicity, was located between the common carotid artery (CCA) and the longus colli muscle (LCM) on the longitudinal ultrasound image.

**Figure 5:**
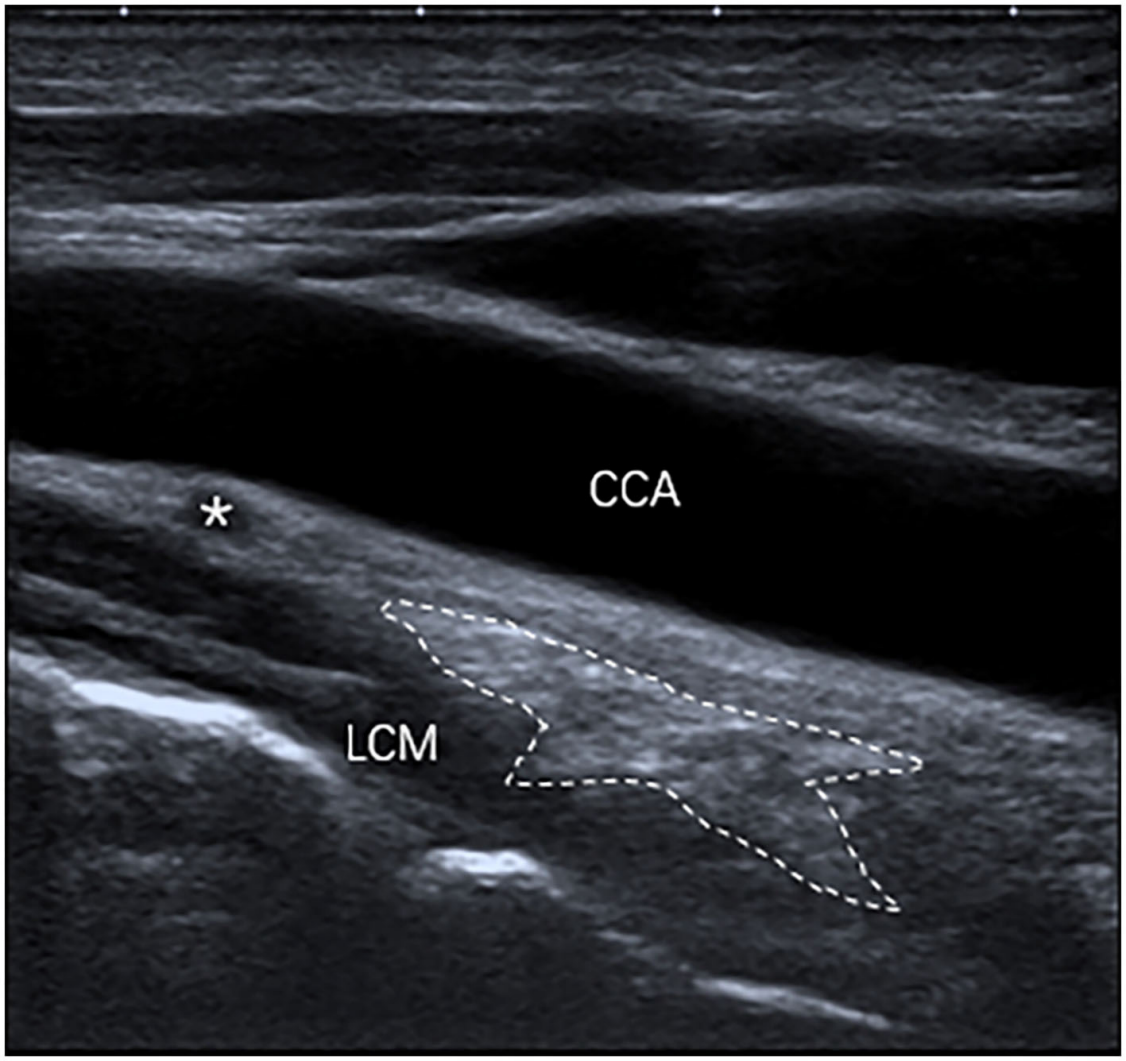
The SG, with a star shape (outlined) and hyperechogenicity, was located between the common carotid artery (CCA) and the longus colli muscle (LCM) and was under the inferior thyroid artery (*) on the longitudinal ultrasound image.

**Figure 6:**
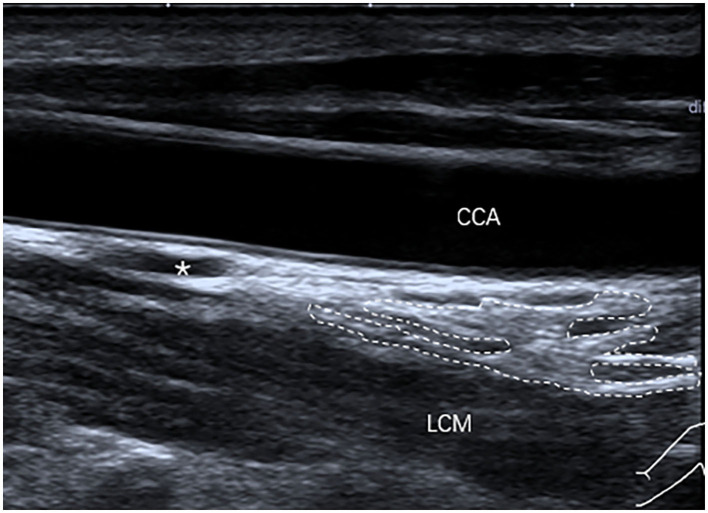
The SG, with a spindle shape (outlined) and hyperechogenicity, was located between the common carotid artery (CCA) and the longus colli muscle (LCM) and was under the inferior thyroid artery (*) on the longitudinal ultrasound image.

The inferior poles of 67 (85%) SGs were visible. The inferior poles of 12 (15%) SGs were not visible. Therefore, the shapes of 67 SGs were assessed, of which 28 (42%) SGs were oval shaped (Figure 2); 18 (27%) SGs were triangular shaped (Figure 3); 14 (21%) SGs were dumbbell shaped (Figure 4); 6 (9%) SGs were star shaped (Figure 5); 1 (1%) SG was spindle shaped (Figure 6). All (100%) SGs showed hyperechogenicity. All (100%) SGs had ill-defined margins. In terms of position, the superior poles of 50 (63%) SGs were located at the C7 transverse process level. The superior poles of 29 (37%) SGs were located behind the C7 transverse process level. The superior poles of 61 (77%) SGs were located under the inferior thyroid artery, of which six SGs were almost close to the inferior thyroid artery. In the remaining 55 (70%) SGs, the distance from the inferior thyroid artery to the superior pole of SG was 10.50 ± 5.83 (2.5–28) mm. Two (3%) SGs were at the same level as the inferior thyroid artery. Sixteen (20%) SGs were not assessed for the relationship with the inferior thyroid artery. All SGs were located lateral to the thyroid and medial to the anterior scalene muscle and the vagus nerve.

## Discussion

In this study, we investigated the US detection rate and characteristics of the SG in a cohort of healthy adults. It has been shown that a majority of SGs can be identified using US. It has also been shown that SG primarily involved hyperechogenicity, ill-defined margin, C7 transverse process level, and under the inferior thyroid artery.

In this study, SG was identified in 79% of the participants. This result was similar to previous studies showing that the incidence of SG ranged from 75 to 80% in most studies (Pather et al., 2006). The favorable result of our study provided evidence for the feasibility of detecting SG by US. We also found that bilateral incidence (61%) was more than that of unilateral side (39%), which was similar to a cadaver study by Pather et al. (2006) who reported that bilateral SG accounted for 65.3%. However, this finding was inconsistent with other cadaver studies by Saylam et al. (2009) and Kommuru et al. (2014). Kommuru et al. (2014) found that four cases were identified bilaterally and fifteen cases were identified unilaterally. Saylam et al. (2009) revealed that all SGs/inferior cervical ganglions were identified bilaterally. On the basis of our clinical study result, the side was associated with SG visibility (*p* < 0.05). SG was more prevalent on the left side compared to the right side (92 vs. 62%). However, the right width was significantly higher than the left. No significant differences were found between the right and left sides regarding thickness and CSA (all *p* > 0.05). These findings suggest that SG visibility may not be related to its size. In the future, further studies should be conducted to validate this finding.

According to the cadaver study, the width, length, and thickness of normal SG ranged from 3.5 to 15.6 mm (Saylam et al., 2009), 5.1 to 25 mm (Saylam et al., 2009; Kastler et al., 2013), and 3.9 to 5 mm (Kiray et al., 2005; Marcer et al., 2012; Kastler et al., 2013), respectively. Our results showed that the width (3.6–7.7 mm) of SG was within the range reported in the literature and that the length (9.8–25.2 mm) of SG was similar to the literature. However, the thickness (1.8–5.3 mm) of some SGs in our study differs slightly from the literature, because the minimum value of the thickness was lower than in the cadaver study. The differences in size may be partly attributed to differences in study cohorts and ill-defined margins of SG on the US images.

SG has a variety of shapes, including star, oval, globular, triangular, spindle, dumbbell, inverted-L, perforated, and truncated forms (Marcer et al., 2012; Kastler et al., 2013; Lee et al., 2017). In our study, SG also displayed various shapes, such as oval, triangular, dumbbell, star, and spindle forms. One reason why other shapes were not found in this study may be that 15% SGs were not assessed because their inferior poles were not visible. Another reason may be that there remains a lack of consensus concerning the categorization of shapes in the related literature reports. For example, a study revealed that Pather et al. (2006) divided other forms reported by other authors into inverted-L forms (Marcer et al., 2012).

The cervical sympathetic nerve is composed of the cervical sympathetic ganglion and the cervical sympathetic trunk. Cervical sympathetic ganglion consists of superior cervical ganglion, middle cervical ganglion, intermediate cervical ganglion or vertebral ganglion, and inferior cervical ganglion or SG. A previous study successfully demonstrated the cervical sympathetic trunk and the middle cervical ganglion by the reconstruction of a three-dimensional ultrasound image, and the cervical sympathetic trunk was confirmed by cadavers, imaging, and clinical signs (Gofeld et al., 2009). Depending on the figure of the reconstruction of the three-dimensional ultrasound, the echogenicity of the cervical sympathetic trunk and the middle sympathetic cervical ganglion were all higher than the LCM. The echogenicity was in agreement with that of SG in our study.

Our study demonstrated that the inferior poles of 15% SGs were not visible, which may be due to the fact that the inferior poles of some SGs were obscured by the bone tissue, so it was difficult to detect with US. When we analyzed the relationship between the superior pole of SG and the inferior thyroid artery, we found that most of the superior pole of SGs (77%) were located under the inferior thyroid artery. This finding may be beneficial to localize the position of SG in the clinic.

The location of the SG was changeable. In our cohort, we divided the position into two types, including the C7 transverse process level and behind the C7 transverse process level. In addition, the superior poles of 63% SGs was located to the C7 transverse process level, and the superior poles of 37% SGs was located to behind the C7 transverse process level. The result was in line with finding in a cadaver study, which showed that the upper pole of the SG was located at the C7 transverse process level in 63.2% of specimens, and the remaining 36.8% of specimens were located between the first rib and the C7 transverse process (Kiray et al., 2005). However, another cadaver study has some differences, revealing that 40% SG/inferior ganglion were located at the level of C7, 25% at the level of C7-Th1 disc, and 35% at the level of Th1 (Saylam et al., 2009). Part of the reason for this relevant discrepancy may be the different study samples.

Admittedly, this study had some limitations. The most important limitation was that the verification of histology concerning SG was not performed. However, since healthy subjects, even patients undergoing the US-guided SGB, usually do not undergo pathological examination, it is difficult to be confirmed by pathology. To reduce the possibility of misdiagnosis, SG was needed to satisfy the following conditions: (1) the nodular structure; (2) between the carotid sheath and the LCM; (3) between the C7 transverse process and the supraclavicular fossa; and (4) found both on the transverse section and the longitudinal section. The efficacy of US-guided SGB by identifying and monitoring SG itself should be further evaluated in future clinical practice.

Despite these limitations, it turned out that most US findings were similar to cadaver studies in our research. In conclusion, our preliminary study demonstrates that US imaging provides the capability of detecting SG. This may be helpful in minimizing complications and improving the accuracy of US-guided SGB.

## Data availability statement

The datasets presented in this article are not readily available because of ethical and privacy restrictions. Requests to access the datasets should be directed to the corresponding authors.

## Ethics statement

The studies involving human participants were reviewed and approved by Shanghai Jiao Tong University Affiliated Sixth People's Hospital. The patients/participants provided their written informed consent to participate in this study. Written informed consent was obtained from the individual(s) for the publication of any potentially identifiable images or data included in this article.

## Author contributions

JL: conceptualization, data curation, formal analysis, investigation, methodology, visualization, and writing-original draft. SP: investigation and visualization. ZL: formal analysis. LJ: methodology, project administration, supervision, validation, and writing—review and editing. YZ: conceptualization, funding acquisition, methodology, project administration, resources, supervision, validation, and writing—review and editing. All authors contributed to the article and approved the submitted version.

## Funding

This work was supported by the Medical Innovation Research Special Key Project of the 2021 Science and Technology Innovation Action Plan in Shanghai (No. 21Y21901100).

## Conflict of interest

The authors declare that the research was conducted in the absence of any commercial or financial relationships that could be construed as a potential conflict of interest.

## Publisher's note

All claims expressed in this article are solely those of the authors and do not necessarily represent those of their affiliated organizations, or those of the publisher, the editors and the reviewers. Any product that may be evaluated in this article, or claim that may be made by its manufacturer, is not guaranteed or endorsed by the publisher.
